# Non‐Invasive Vagus Nerve Stimulation in Cluster Headache: A Clinical Practice Guideline

**DOI:** 10.1111/papr.70084

**Published:** 2025-10-24

**Authors:** Peter J. Goadsby, Alexander Feoktistov, Magdalena Anitescu, Miles Day, Peter Staats

**Affiliations:** ^1^ NIHR King's Clinical Research Facility and Wolfson SPARC King's College London London UK; ^2^ Department of Neurology University of California Los Angeles California USA; ^3^ Synergy Integrative Headache Center Chicago Illinois USA; ^4^ American Interventional Headache Society Bath Ohio USA; ^5^ Neurology Department of Clinical Sciences Rosalind Franklin University of Medicine and Science North Chicago Illinois USA; ^6^ Department of Anesthesia and Critical Care Medicine University of Chicago Medicine Chicago Illinois USA; ^7^ Department of Anesthesiology Texas Tech University Health Sciences Center Lubbock Texas USA; ^8^ Vagus Nerve Society Atlantic Beach Florida USA

**Keywords:** bioelectronic medicine, cluster headache, headache management, neuromodulation, tcVNS, vagus nerve stimulation

## Abstract

**Background:**

Cluster headache (CH) is a rare but severe primary headache disorder characterized by recurrent attacks of unilateral, typically periocular pain lasting 15 min to 3 h, accompanied by ipsilateral autonomic symptoms and restlessness or agitation. Attacks may occur multiple times daily and present in clusters lasting weeks to months, interspersed with remission periods in episodic CH, or without remission in chronic CH.

**Methods:**

This review summarizes the clinical evidence supporting the use of transcutaneous cervical vagus nerve stimulation (tcVNS) for both the acute and preventive treatment of CH. Relevant clinical trials, real‐world studies, and guideline recommendations are discussed.

**Results:**

Pharmacological therapy for CH includes triptans and high‐flow oxygen for acute management, and verapamil, corticosteroids, or galcanezumab for prevention. For patients with inadequate response or intolerance to these options, neuromodulation may be required. TcVNS has emerged as a noninvasive, safe, and effective alternative to invasive neuromodulation. Clinical trials have demonstrated significant reductions in attack frequency and intensity, leading to U.S. Food and Drug Administration (FDA) clearance and UK National Institute for Health and Care Excellence (NICE) approval for both acute and preventive treatment of CH.

**Conclusions:**

TcVNS represents a well‐tolerated, noninvasive neuromodulatory option for patients with cluster headache, offering both acute and preventive benefits. This paper provides an overview of the current evidence, mechanisms of action, and practical guidelines for incorporating tcVNS into clinical management.

## 1 Introduction

If you want to understand what it means to live with cluster headache, imagine that someone is stabbing a knife in your eye and turning it for hours. Imagine the worst pain. Imagine a daily torture, gratuitous, incomprehensible. Imagine yourself suffering alone, terribly. Imagine being a prisoner in a straitjacket of suffering… Imagine the desire to finish, with pain, and the desire to finish… with yourself. If you imagine, you will understand.

—Thomas, a patient with cluster headache [1]

Cluster headache (CH) is a primary headache disorder and the most common of the trigeminal autonomic cephalalgias [2]. CH is a debilitating condition with pain that has been described as worse than gunshot wounds, childbirth, kidney stones, or pancreatitis [3, 4]. The intensity of the pain and frequency of the attacks are so unbearable that 55% of patients are driven to consider suicide, earning CH the moniker, “suicide headache” [1, 5]. Longer disease duration is associated with an overall increase in suicidal ideation and attempts [6].

The International Classification of Headache Disorders (3rd edition) (ICHD) defines CH as attacks of sudden‐onset unilateral pain in the supraorbital, orbital, or temporal region, lasting 15 min to 3 h and occurring at a frequency of once every other day to up to 8 times a day. Attacks occur in “cluster periods” or “bouts,” which last from 7 days to a year and are separated by remission periods of at least 3 months. The headaches are accompanied by restlessness, agitation, and/or one or more of the following cranial autonomic symptoms ipsilateral to the headache: conjunctival injection and/or lacrimation, miosis and/or ptosis, nasal congestion and/or rhinorrhea, eyelid edema, and/or diaphoresis of forehead and face [7]. The pain of CH is positively correlated with the frequency of the attacks, rather than the duration of each attack, and with the presence of cranial autonomic features [4]. Up to 15% of patients with CH experience chronic CH (CCH), defined by the ICHD as CH lasting ≥ 1 year, either without remission or with remission lasting less than 3 months [7].

## 2 Epidemiology and Diagnosis

The prevalence of CH is approximately 0.1%, with a mean age of onset of 30 ± 14 years [8]. Men are presumed to be affected more than women by a ratio of approximately 3:1. This distribution is a subject of discussion, with evidence that women are affected at much higher rates than previously recognized [7, 9]. Data on CH in non‐Caucasian populations is limited, but evidence suggests that the epidemiology and phenotype of CH differ by race. For example, one study found that the incidence of CH is higher in African American women than in African American men [8]. In contrast, in Asian populations, the incidence is higher in males, and restlessness is less common compared to European and North American populations [10].

The diagnosis of CH is based on clinical presentation and the exclusion of other primary and secondary headache disorders [2]. There are no serum markers or single gene polymorphisms that definitively point to CH, and although MRI images show multiple abnormalities in CH, there are no conclusive findings on imaging studies that would confirm a diagnosis [11, 12]. Diagnostic delays are common, as only 1 in 5 patients receive a correct diagnosis at the time of initial presentation, and most receive multiple diagnoses before they are determined to have CH [2, 5, 13]. The average time from first symptoms to accurate diagnosis is 5–9 years. Misdiagnosis is especially common in women, both because of the implicit bias that CH is a disease of men and also because CH in women presents with more migraine‐associated symptoms [9, 14].

Risk factors include a family history of CH (evidence supports both autosomal dominant and recessive patterns of inheritance) [15], smoking or illicit drug use, and a history of traumatic brain injury (TBI) [8, 12]. Common comorbidities include depression and sleep disorders [2]. Although CH is considered a rare headache disorder, it places a disproportionately high socioeconomic burden on individuals, families, the healthcare system, and society as a whole. It is a chronically disabling condition affecting employment, self‐worth, parenting, and comorbidities such as depression and anxiety, even during remission [8, 16, 17].

## 3 Pathophysiology

The pathophysiology of CH is incompletely understood but is known to involve the trigeminovascular system, the trigeminal‐autonomic reflex, and the hypothalamus [8]. The trigeminovascular system consists of an extensive network of neurons within the cranium from the dura mater and meninges to the thalamus via the trigemino‐cervical complex. Activation of the trigeminovascular system causes the release of neuropeptides, such as calcitonin gene‐related peptide (CGRP), which mediates nociception [18, 19]. The second‐order neurons in the trigeminovascular system that are activated during the pain response also trigger the trigeminal‐autonomic reflex, causing parasympathetic activation resulting in lacrimation, conjunctival injection, and nasal congestion and sympathetic inhibition, causing ptosis and miosis [20]. In addition, the trigeminovascular system connects with the hypothalamus, which contributes to nociception as well as controlling circadian, neuroendocrine, and autonomic regulation [2, 21]. Physical evidence that the hypothalamus is involved includes the circannual and circadian patterns of CH attacks and neurohormonal changes in testosterone, melatonin, and cortisol experienced by patients with CH [22, 23].

## 4 Management

Pharmaceutical treatment options for CH fall into four categories: acute, bridge, preventive, and refractory. First‐line acute abortive therapies include sumatriptan (subcutaneous [24] or intranasal [25]), intranasal zolmitriptan [26, 27], and high‐flow oxygen [28]. While effective, triptans have their drawbacks, as they have vasoconstrictive effects and are therefore contraindicated in patients with cardiovascular disease.

Bridging therapy is used while transitioning to preventive therapy or as short‐term prophylaxis. The two standard forms of bridging therapy for CH are prednisone 100 mg/d for 5 days with a taper and a greater occipital nerve block [29, 30].

The most effective preventive medications include verapamil and lithium, with galcanezumab recently approved for episodic cluster headaches. Other options like topiramate and corticosteroids may also be considered, especially when first‐line treatments are ineffective or contraindicated [30]. Patients on verapamil or lithium need regular monitoring for cardiovascular side effects as well as electrolytes, thyroid function, and drug levels (in the case of lithium) [8, 31].

Many will target multiple levels of treatment and prevention at the same time for their patients with CH. For example, a single patient may be prescribed a triptan plus high‐flow oxygen therapy, a bridge treatment (such as an occipital nerve block), and a preventive medication (such as verapamil) [32].

Neuromodulation is an excellent option for patients in whom pharmacological agents are contraindicated or for the treatment of those who are medically refractory. Invasive interventions, such as sphenopalatine ganglion [33] or greater occipital nerve stimulation [34], are effective, costly, and carry surgical risks [8, 31, 35]. In contrast, multiple studies and real‐world data have shown that transcutaneous cervical vagus nerve stimulation (tcVNS) is safe and effective for the acute, preventive, and refractory treatment of CH [36, 37].

## 5 Vagus Nerve Stimulation

### 5.1 Anatomy and Physiology

The vagus nerve is essential to regulating and maintaining homeostasis. The longest and most wide‐ranging of the cranial nerves, the vagus acts as a superhighway connecting the body and the brain. It is made up of approximately 80% unmyelinated sensory afferent fibers and 20% myelinated motor efferent fibers [38, 39]. Its path through the neck is posterior to the carotid artery and medial to the internal jugular vein. Although the mechanism is incompletely understood, there are extensive interconnections between vagal fibers and the neurons of the trigeminal–thalamocortical pathways; therefore, stimulation of the vagus can have multiple neuropsychological and neurophysiological effects, including the regulation of pain, inflammation, and mood [40, 41]. The vagus nerve connects with the trigeminal system at the rostral medulla, where vagus fibers intersect with the trigeminal spinal nucleus and the trigeminal spinal tract [42]. Vagus nerve stimulation reduces dural‐evoked and spontaneous trigeminocervical neurons with nociceptive inputs [43] through a mechanism that at least has the involvement of the delta opioid receptor [44].

Preclinical studies support the use of tcVNS in the treatment of primary headaches, including migraine and cluster headache. Animal studies have shown that tcVNS can help with cluster headaches by:

- Reducing nociception: In rats with chronic allodynia, tcVNS reduced pain and trigeminal allodynia for 3.5 h. Improvement was associated with a block or reverse of high glutamate levels in the trigeminal nucleus caudalis (TNC) and suppression of extracellular glutamate levels. GABA, glycine, norepinephrine, and 5‐hydroxytryptamine (5‐HT) levels stayed unchanged [45].
- Reducing neuron activation: In validated animal models of acute dural‐intracranial (migraine‐like) and trigeminal‐autonomic (cluster‐like) head pain, tcVNS suppressed pain‐induced firing of trigeminocervical neurons [43, 44].
- Enhancing pain modulation: tcVNS inhibited trigeminal activation via the involvement of GABAergic and serotonergic signaling [46]
- Suppressing pain development and maintenance: inhibited mechanical nociception and repressed expression of proteins associated with peripheral and central sensitization of trigeminal neurons in a novel rodent model [47]

### 5.2 Clinical Findings

Initial evidence that stimulation of the vagus nerve could benefit patients with primary headache disorders came from case reports and small studies of patients who were treated with surgically implanted VNS devices for other indications [48, 49, 50]. Neuromodulation of the vagus using an implanted device, traditional VNS, was approved by the Food and Drug Administration (FDA) for the treatment of drug‐resistant epilepsy, treatment‐resistant depression, and post‐ischemic stroke rehabilitation for over 20 years. The traditional VNS device consists of a small electrical generator inserted into the subcutaneous tissue of the left anterior chest, an electrode cuff around the left cervical vagus nerve, and a wire connecting the two. The device is then programmed to deliver intermittent impulses at a frequency and intensity that is adjusted by a neurologist or psychiatrist [51].

Adverse events associated with traditional VNS include the surgical risks of infection, hematoma, vocal cord palsy, voice alteration, dyspnea, neck pain, and the ongoing need for further intervention for battery and lead replacements [52]. Constant intermittent stimulation can cause voice alterations, paresthesias, cough, and headache [53]. For patients with CH, traditional VNS can be risky, cost‐prohibitive, and challenging to access.

The latest advances in vagus nerve stimulation include two forms of non‐invasive VNS: transcutaneous cervical VNS (tcVNS), applied over the anterolateral neck, and transcutaneous auricular VNS (taVNS), applied to the concha or tragus of the ear [50, 54]. Functional magnetic resonance imaging (fMRI) reveals similar regional brain activation with non‐invasive VNS and traditional VNS, indicating that the two methods have comparable mechanisms [55]. Non‐invasive VNS has several advantages over traditional VNS, including a better safety profile, the lack of surgical side effects, improved tolerability, lower cost, and greater accessibility. In addition, the activation of non‐invasive VNS devices is user‐controlled, while traditional VNS provides constant on–off pulses, so the number and severity of stimulation‐related adverse events are much lower with non‐invasive VNS. Notably, the ease of use and accessibility of non‐invasive VNS might make it an option that can be considered earlier in the disease course, so patients do not have to wait until their CH is considered “treatment‐refractory”.

TaVNS is currently being evaluated in clinical trials for use in disorders of consciousness, such as impaired awareness resulting from TBI, stroke, or hypoxic–ischemic encephalopathy [56]. It is also being evaluated for multiple other applications, including depression, gastrointestinal conditions, addictions, and motor rehabilitation [57].

TcVNS is primarily used for migraine and cluster headache and is also being evaluated for multiple other indications, including other pain disorders, inflammatory, cardiovascular, and gastrointestinal diseases, as well as learning, focus, and performance enhancement, and fatigue mitigation [50, 58]. In 2011, gammaCore received a CE (Conformité Européene) mark in the European Union for primary headache. gammaCore was cleared by the FDA for the treatment of eCH in 2017 and for the prevention of CH in 2018 [59].

The only FDA‐cleared and most widely used tcVNS device is gammaCore, a small, rechargeable unit that generates, amplifies, and transmits a low‐voltage electrical signal through two stainless steel stimulation surfaces that contact the skin over the anterior neck in the area of the vagus nerve. This device produces a proprietary electrical signal consisting of five 5000‐Hz sinusoidal pulses each of 200 μs at a frequency of 25 Hz, with a peak voltage of 24 V and a maximum output current of 60 mA. The user can adjust the amplitude through simple controls on the unit [60].

The patient applies conductive gel to the stimulation surfaces, turns on the device, and places the stimulation surfaces over the cervical vagus nerve for two consecutive 2‐min stimulations, which are repeated, depending on the health care provider's directions. If the headache fails to abort, the process can be repeated. gammaCore can be used for a total of 24 stimulations per day. For the prevention of chronic CH, gammaCore is administered for two consecutive 2‐min stimulations two to three times a day [61].

## 6 TcVNS in CH

The safety and tolerability of tcVNS are consistent throughout clinical studies and reports of real‐world experience. The most common side effects are perioral muscle contraction during treatment, skin irritation or tingling, burning sensations, muscle pain, and/or redness at the application site. No serious device‐related adverse events have been reported, and there are no side effects to warrant safety monitoring [62].

Two large, multicenter, randomized, double‐blind, sham‐controlled clinical trials, ACT1 (ACute Treatment of cluster headache) and ACT2, and a meta‐analysis of the pooled data from both trials provided evidence supporting the efficacy of tcVNS for the acute treatment of episodic CH [63, 64, 65]. Among patients with episodic cluster headache, response rates for the first treated attack were significantly higher in the tcVNS group than in the sham cohort in ACT1 (34% vs. 11%; *p* = 0.008) and in the pooled ACT1/ACT2 populations (39% vs. 12%; *p* < 0.01). Response was defined as a reduction of pain intensity to 0 or 1 on a 5‐point scale 15 min after treatment initiation without the use of rescue medication. The proportion of all treated attacks that had pain‐free status at 15 min was significantly greater among tcVNS patients than among sham‐treated patients for the ACT1 (15% vs. 6%; *p* < 0.05), ACT2 (48% vs. 6%; *p* < 0.01), and pooled ACT1/ACT2 populations (24% vs. 7%; *p* < 0.01).

Evidence supporting the efficacy of tcVNS for the prevention of CH was provided by the randomized, controlled PREVention and Acute treatment of chronic cluster headache (PREVA) trial [61, 66]. In this trial, tcVNS plus the standard of care (SoC) was associated with a significantly greater reduction in the number of cluster attacks per week versus SoC alone (−5.9 vs. –2.1; *p* = 0.02). Tc‐VNS plus SoC also resulted in significantly higher ≥ 50% response rates than SoC alone (40% vs. 8%; *p* < 0.001). The response rate of ≥ 50% was defined as the proportion of patients with a ≥ 50% reduction in the mean number of CH attacks per week. Tc‐VNS plus SoC also led to a significant decrease (57%; *p* < 0.001) in the frequency of abortive medication use. A post hoc analysis of the PREVA study confirmed a sustained reduction in CH attack frequency with prophylactic tcVNS. See Table 1.

**TABLE 1:** Randomized clinical trials of tcVNS for cluster headache.

Study name	Cluster headache type	No. of patients (ITT)	Study design	Primary findings	Publication
*Preventive treatment of cluster headache*					
PREVA	Chronic Cluster Headache	93	Multicenter, open‐ label, randomized controlled	5.9 fewer attacks per week in nVNS + SoC group vs. 2.1 fewer attacks in SoC alone	Gaul C, et al. Cephalalgia, 2015
				Significant reduction in chronic CH attack frequency by week two and a significant decrease in the use of acute medications	Gaul C, et al. J Headache Pain, 2017
				nVNS was cost‐effective compared with current SoC	Morris J, et al. J Headache Pain, 2017
*Acute treatment of cluster headache*					
ACT1	Episodic cluster headache/Chronic cluster headache	133	Multicenter, randomized, double‐ blind, sham‐controlled	Significant pain reduction at 15 min for episodic CH	Silberstein SD, et al. Headache, 2016
ACT2	Episodic cluster headache/Chronic cluster headache	92	Multicenter, randomized, double‐ blind, sham‐controlled	Pain freedom at 15 min for episodic CH subgroup	Goadsby PJ, et al. Cephalalgia, 2018
ACT1/ACT2 Pooled	Episodic cluster headache/Chronic cluster headache	225 (ACT 1/ACT 2 pooled)	Pooled analysis	Pain‐free status at 15 min was significantly greater within nVNS	de Coo, IF, et al. Cephalalgia, 2019

Abbreviations: CH, cluster headache; ITT, “intention‐to‐treat”; every individual randomized to a treatment group is analyzed as part of that group; nVNS, non‐invasive vagus nerve stimulation; SoC, standard of care.

Analyses of real‐world data from patients with CH support these findings. In one retrospective study, patients with CH, most of whom were unresponsive to or intolerant of multiple acute and preventive treatments, experienced significant reductions in the frequency, severity, and duration of CH attacks with tcVNS and were able to decrease their medication use [67]. An open‐label observational study of 40 patients with refractory chronic CH found that patients who received tcVNS experienced a significant reduction in attack frequency (*p* < 0.001) and severity (*p* = 0.001) [68]. Another observational study in patients with multiple morbidities found that tcVNS significantly improved quality of life (QoL) and lowered healthcare costs, and a significant number of patients remained adherent to the tcVNS regimen, supporting its tolerability [69]. Data from a voluntary tcVNS patient registry (GPR) provided by patients with episodic CH showed that in the 70% of attacks that were treated with tcVNS, the pain was reduced to 0 to 1 on a 5‐point scale by 30 min after initiation of treatment, supporting the findings of the ACT1 and ACT2 trials [70]. A study of prescribing and refill trends in the United Kingdom found that patients who were prescribed tcVNS for CH continued to request refills through multiple 3‐month refill cycles, indicating their satisfaction with the safety and tolerability of the device [71].

## 7 Economic Benefit

The economic burden of cluster headache is considerable, costing more than twice that of non‐headache patients. In a cost‐effectiveness analysis reported by Morris et al. [72] based on a subset of PREVA study data from Germany, gammaCore + standard of care (SOC) was cost‐saving compared with SOC alone from the German payer perspective. In another cost‐effectiveness analysis based on a subset of PREVA study data from the United Kingdom, Jenks et al. [73] found the incremental cost‐effectiveness ratio (ICER) from the UK payer perspective was £13,368 per quality‐adjusted life‐year gained when comparing gammaCore + SOC with SOC alone. Mwamburi et al. conducted a cost‐effectiveness analysis of gammaCore adjunct to SOC compared with SOC alone for the treatment of acute pain associated with episodic cluster headache attacks. The gammaCore + SOC arm was dominant over SOC alone [74].

## 8 Patient Selection/Screening

Candidates for treatment with tcVNS include patients ≥ 18 years of age with either episodic or chronic CH for whom SoC is insufficiently effective, poorly tolerated, or contraindicated, and who are willing to use a neuromodulation device. tcVNS does not replace pharmacological treatments for CH but is used in addition to them and can result in reduced medication use [36]. tcVNS is contraindicated for patients with an active implanted device near their neck, such as a pacemaker or hearing aid implant. The device should not be used if the patient is using another electronic device, such as a TENS unit, at the same time. gammaCore should also not be used near microwaves or medical imaging machines. The device should not be used on wet skin or near an open wound, rash, infection, drug patch, or surgical scar.

Adequate training on how to use the device is essential for patient success. Training is simple and can be completed in one session [75]. Patients are instructed to remove any jewelry close to the treatment site, locate the carotid pulse to identify the treatment location, apply a small amount of electrolyte gel to the stimulation surfaces, turn the device on by pressing the power button, position the device over the carotid pulse with mild to moderate pressure, increase the intensity (on a scale of 1–40) to a level that causes muscle contractions at the corner of the mouth by pressing a control button on the side of the device, and deliver the treatment. Most patients use an intensity averaging between 15 and 25. The device will automatically beep twice and stop delivering stimulation after 2 min. A display on the device shows the number of stimulations and days remaining and a record of the last intensity level achieved [76].

Since the response to tcVNS varies among individuals, patients should be reassessed after 3 months of use, and the device may be discontinued if there is no effect within 3 months. Patients should be re‐evaluated 12 months after the initiation of treatment and once a year after that. During follow‐up visits, assessments should include measures of subjective and objective improvement, the rate of adverse events, the use of rescue medication, QoL parameters, frequency of use, and effect on comorbid conditions [76].

## 9 Discussion/Conclusion

Multiple clinical trials, observational studies, retrospective studies, and case reports support the use of the FDA‐cleared tcVNS device for the treatment of episodic CH and the prevention of chronic CH when used in conjunction with SoC. The excellent safety profile and high tolerability of the device result in high rates of adherence. Patients with CH who respond to tcVNS experience additional benefits beyond symptomatic improvement, such as a decreased need for medication, avoidance of invasive procedures, reduced healthcare costs, and better QoL. There is an ongoing need for further research to clarify the pathophysiology of CH and the mechanism of action of VNS, to identify characteristics of responders and non‐responders, and to assess the possible long‐term benefits of tcVNS. Nevertheless, the ease of use, the safety profile, and the evidence to date make this technology a highly accessible and effective option for patients who suffer from one of the most painful illnesses imaginable.

## Author Contributions

P.S. conceived and outlined the paper. All authors discussed the draft and contributed portions of text to the final manuscript. All listed authors have agreed to the final submitted version.

## Ethics Statement

The authors have nothing to report.

## Consent

The authors have nothing to report.

## Conflicts of Interest

Dr. Peter Staats is an Editorial Board member of “Pain Practice” and a co‐author of this article. To minimize bias, he was excluded from all editorial decision‐making related to the acceptance of this article for publication. Dr. Staats is also employed by electroCore Inc. and holds stock in the company. Remaining authors do not have any potential conflicts of interest to disclose.

## Data Availability

The authors have nothing to report.
